# Ileorectal anastomosis in ulcerative colitis: what do surgeons and patients need to know? A systematic literature review

**DOI:** 10.1308/rcsann.2024.0012

**Published:** 2024-04-25

**Authors:** MR Orchard, A Saracino, J Hooper, J Shabbir

**Affiliations:** ^1^ University Hospitals Bristol and Weston NHS Foundation Trust, UK; ^2^ Somerset NHS Foundation Trust, UK

**Keywords:** ulcerative colitis, ileorectal anastomosis

## Abstract

**Introduction:**

Ileal pouch–anal anastomosis (IPAA) is currently the gold standard for restoration of gastrointestinal continuity after colectomy for ulcerative colitis in the UK. However, with further experience of the risks relating to IPAA, the use of ileorectal anastomosis (IRA) is being revisited. Decisions regarding restorative surgery after colectomy are individual to every patient's circumstances, and this paper aims to provide a comprehensive review of the literature to guide a full discussion of the risks and benefits of IRA.

**Methods:**

A systematic literature review was conducted of papers published from 2000 onwards relating to IRA and ulcerative colitis, in accordance with the PRISMA (Preferred Reporting Items for Systematic reviews and Meta-Analyses) guidelines. The papers were reviewed by two independent surgeons for information it was felt that patients and surgeons would want to know about the operation (cancer risk, bowel function, sexual and urinary function, fecundity/fertility and postoperative complications).

**Results:**

Seventeen papers were identified for inclusion as they reported original data on one or more of the categories identified for discussion. The median ten-year cancer risk after IRA was 2.8% and the median failure rate at ten years was 21%. IRA was generally found to have lower postoperative complication rates and better bowel function than IPAA, with sexual function similar and fecundity not commented on in any paper.

**Conclusions:**

For some patients, IRA can offer restorative surgery in the short or long term, with acceptable cancer risk, failure rate and postoperative complications, while avoiding the higher risks associated with IPAA.

## Introduction

The proportion of patients requiring colectomy for acute severe ulcerative colitis (UC) has been estimated at approximately 10% at ten years after diagnosis.^1^ In the UK, a third of patients undergo further reconstructive surgery.^2^ The decisions around reconstructive surgery remain complex and very individual for patients. As surgeons, we should provide them with a full discussion of the risks and benefits of all the options: no further surgery, completion proctectomy with permanent ileostomy, ileal pouch–anal anastomosis (IPAA) or ileorectal anastomosis (IRA).^3^

IRA was first described in the 1950s and was the first operation that gave restoration of gastrointestinal continuity for patients after colectomy.^4^ Despite its popularity in some centres, concerns were raised about the risk of anastomotic leak, cancer developing in the remaining rectum and high long-term failure rates due to persistent proctitis.^5^ IRA is also not appropriate for all patients. Those with severe proctitis and a non-compliant rectum are unlikely to have good function and IRA is not recommended.^6,7^

With the advent of the IPAA in the 1980s, IRA fell out of favour in the UK and IPAA has now become the gold standard operation for these patients.^8^ Between 2002 and 2012, 92.3% of patients who underwent reconstruction after colectomy for UC in the UK opted for IPAA, compared with 7.7% for IRA.^2^ Interestingly, IRA remained popular in some countries, with widespread use particularly in Sweden, where 59% chose IRA compared with 39% opting for IPAA.^2^

With further experience of IPAA, it is clear that it is a technically rigorous operation with potentially significant postoperative risks best managed in specialist centres.^9^ Pouch surgery has known effects on fecundity, poses risk to pelvic nerves and does not fully eliminate the rectal cancer risk.^8^ Patients can also suffer with chronic pouchitis, chronic pelvic sepsis and poor function.^10^

With difficult decision making, we owe it to our patients to lead a fully informed discussion regarding all the options. With this literature review, we sought to identify the key information that patients and surgeons will need in their shared decision making about IRA, used either as a bridge to a pouch or stoma, or as a long-term option. Similar data for the alternative management options should also be available for discussion but are outside the scope of this review.

## Methods

A systematic literature review was conducted using the Embase^®^, MEDLINE^®^, PubMed^®^, AMED (Allied and Complementary Medicine Database), British Nursing Index, CINAHL^®^ (Cumulative Index to Nursing and Allied Health Literature), Health Business Elite, Healthcare Management Information Consortium and PsycInfo^®^ databases, and in accordance with the 2020 PRISMA (Preferred Reporting Items for Systematic reviews and Meta-Analyses) guidelines.^11^ The search terms employed were “ulcerative colitis” OR “UC” AND “ileo-rectal” OR “ileorectal” OR “IRA”. The primary objective was to assess the cancer risk following an IRA. The secondary objectives were to identify other information regarding postoperative complications and quality of life that would be important to a surgeon or patient considering IRA.

**Figure 1:**
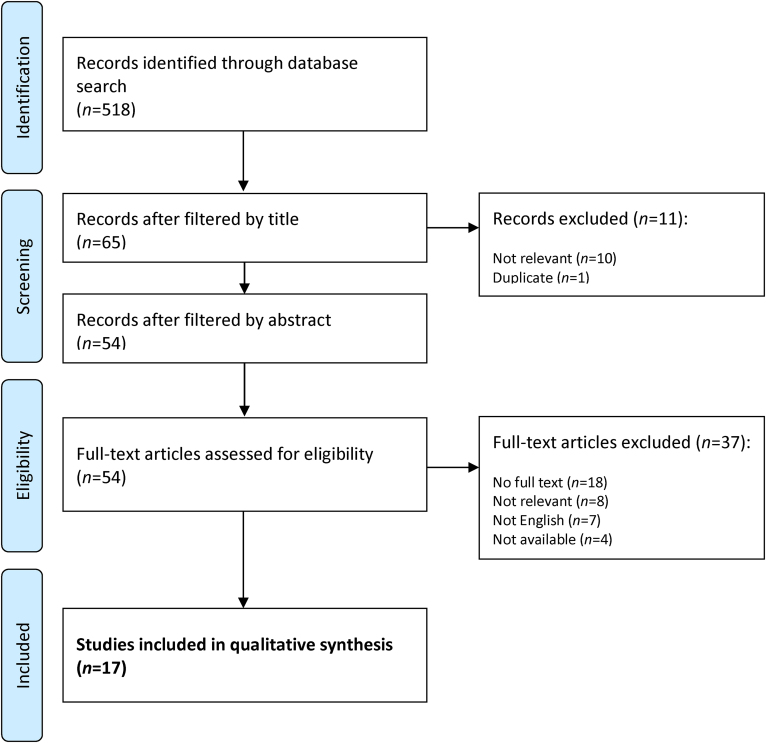
Flowchart of study selection

Our search was limited to papers published after 2000 so as to better represent the modern management of surgical patients and the laparoscopic era. The initial search identified 518 papers published between 2000 and 2022 (Figure 1). These were all reviewed for relevance by title and then by abstract by two surgeons independently. Any papers that gave results relating to cancer risk, postoperative complications or quality-of-life metrics were considered for inclusion, leaving 54 papers for full-text review. Thirty-seven further papers were excluded at this stage. Consequently, this left 17 studies for inclusion in this systematic review.^6,7,12–26^ The same two surgeons manually extracted data from these papers on cancer risk, bowel function, sexual and urinary function, fecundity/fertility and postoperative complications. Where data were given in timescales that were not at five-year intervals, the time was rounded up or down to the nearest five years.

## Results

### Cancer risk

One of the advantages of IPAA over IRA is that it significantly reduces the long-term cancer risk by removing all but a cuff of rectum. This remains one of the primary considerations for surgeons and patients.

**Figure 2:**
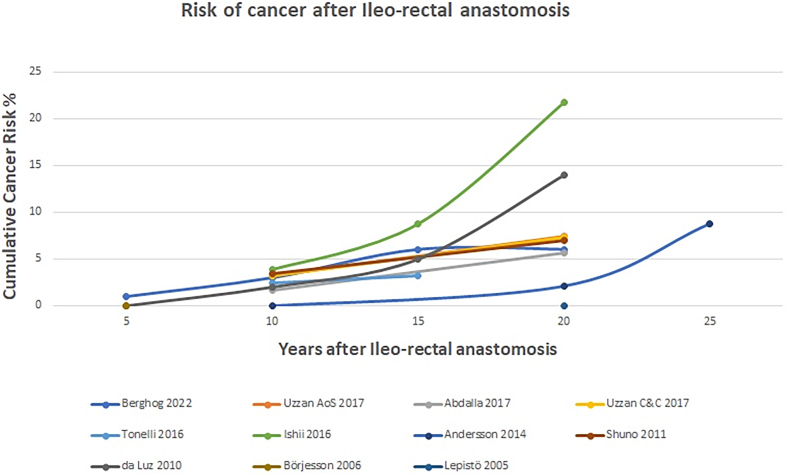
Risk of cancer after ileorectal anastomosis

Eleven papers discussed the risk of developing malignancy in the rectum after IRA.^6,7,12,14,16,18,20,22–25^ All papers noted a cancer risk of <5% at ten years, with a median of 2.8% (Figure 2). This is lower than observed historically but in keeping with a meta-analysis published in 2023.^27^ At 20 years, there was a much larger range for the reported risks: 0–21.7% (median: 7.3%). Figure 2 also illustrates that the cancer risk increases exponentially at 10–15 years after the operation. This fits with the known baseline risk of colorectal cancer in UC, which is reported to be 2% by 10 years’ disease duration, 8% by 20 years and 18% by 30 years.^28^ These cancer risks for IRA patients are higher than those after IPAA, with European guidelines quoting 1.1%^8^ and a 2014 systematic review giving an overall risk of 0.35% for pouch patients.^29^

The cancer risk does vary considerably between different patient groups, however, and this should be borne in mind when deciding whether IRA is the right option for a specific patient. The presence of dysplasia or malignancy in the resected colon is a very significant prognostic marker for development of future rectal cancer. In a retrospective cohort study, at ten years after the operation, rectal cancer occurred in 1.4% of patients if dysplasia or malignancy was not present in the resected colon; this compared with 22.8% if it had been present (*p*=0.0002).^24^ Other factors known to increase the risk of developing rectal cancer following colectomy for UC are the presence of primary sclerosing cholangitis (PSC),^13^ older age and >10 years since UC diagnosis.

Given the ongoing cancer risks, annual surveillance with flexible sigmoidoscopy is recommended following IRA.^6,30^ The importance of surveillance is highlighted by the results from Tonelli *et al*, in which the three IRA patients (2.4%) who had proctectomy and IPAA for high-grade dysplasia had undergone regular surveillance whereas the four (3.2%) who presented with T3/T4 rectal cancers had not.^23^

### Failure rate

**Figure 3:**
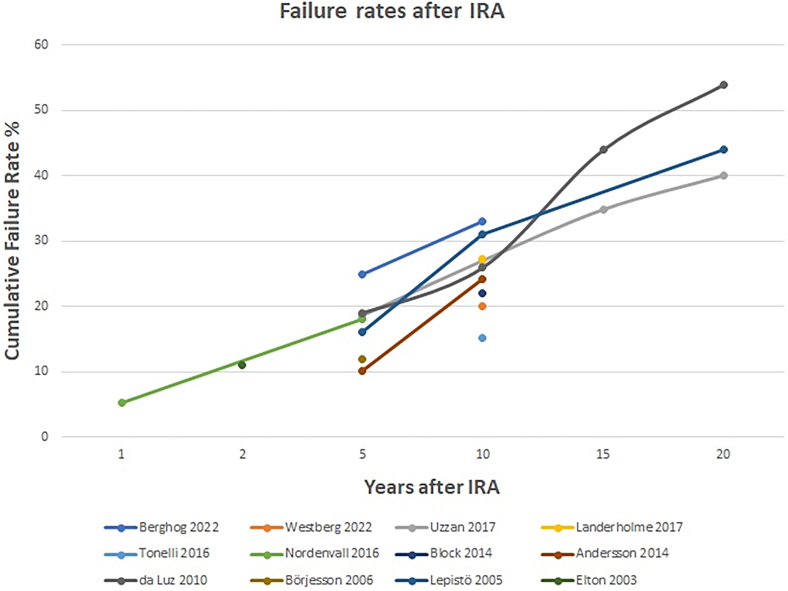
Failure rates after ileorectal anastomosis (IRA)

Historically, one of the criticisms of the IRA was a high failure rate, usually due to cancer or intractable proctitis. Failure is defined as the need for a defunctioning ileostomy or completion proctectomy. Twelve papers in our review discussed failure rates.^6,7,14–17,19–21,23,24,26^ Figure 3 shows the failure rates given in each paper, with a median of 21% (range: 15–33%) at ten years. For comparison, the same papers noted a median failure rate of 13% for IPAA at ten years and a long-term follow-up from the ileal pouch registry demonstrated a 7.7% failure rate.^31^

Once again, there are specific factors to consider for individual patients that help us to guide decision making. Patients with PSC as well as UC (PSC-UC) have higher failure rates after IRA (and IPAA) than those with UC only. This was reported by Block *et al*, with failure rates at 6–8 years after IRA of 53% in PSC-UC patients compared with 22% in those with UC only.^15^ Likewise, Berghog *et al* noted that 18% of patients in their cohort with failure following IRA had PSC-UC compared with 6% having PSC-UC in the non-failure cohort.^6^

### Bowel function

From the patients' perspective, one of the most important factors affecting quality of life is expected function. No papers reported on urinary function and seven papers commented on bowel function.^7,13–17,23^ The results are summarised in Table 1. IRA scored consistently better than IPAA in terms of median bowel movements, incontinence/seepage and functional score but worse for urgency.

**Table 1:** Bowel function after IRA and IPAA

Study	Patients	Median bowel frequency/24h		Incontinence/seepage		Urgency		Median Oresland functional score^a^	
		IRA	IPAA	IRA	IPAA	IRA	IPAA	IRA	IPAA
da Luz Moreira, 2010^7^	86 IRA	6 (range: 2–11)	7 (range: 3–18)	5% (night)	32% (night)	68%	21%		
Abdalla, 2020^13^	38 IRA, 39 IPAA	5 (range: 1–13)	7 (range: 3–14)	7.9%	30.8%	34.2%	15.4%	3 (IQR: 2–5)	10 (IQR: 5–15)
Andersson, 2014^14^	105 IRA, 148 IPAA	4 (range: 1–11)	5 (range: 2–13)						
Block, 2014^15^	17 IRA, 31 IPAA							3 (8% scored ≥8)	5 (12% scored ≥8)
Börjesson, 2006^16^	32 IRA	55% 0–5; 30% 6–8; 15% >8	Worse than IRA (not quantified)	14.8%	Worse than IRA (not quantified)	33%	Worse than IRA (not quantified)		
Elton, 2003^17^	215 IRA (18 UC)^b^	4		10%					
Tonelli, 2016^23^	126 IRA			0% (day), 6% (night)	6.1% (day), 25.5% (night)				
IPAA = ileal pouch–anal anastomosis; IQR = interquartile range; IRA = ileorectal anastomosis; UC = ulcerative colitis									

^a^Oresland scale: 0–15, 15 worst, ≥8 represents reduced quality of life

^b^Others: familial adenomatous polyposis, Crohn's disease, cancer or functional

Alongside this bowel function is the need for ongoing use of medication, which is high in both groups but generally higher following IRA. This is expected as the rectum can still have active proctitis even if it appeared normal preoperatively.^32^ Medication use in patients who had undergone IRA was recorded in seven papers,^7,13,14,16,17,20,23^ including use of topical or systemic treatment for UC (range: 20–91% of patients)^7,14,16,20,23^ and antidiarrhoeal medication (range: 16–60% of patients),^7,16,17,23^ with one paper reporting 90% of patients requiring antibiotics.^23^ Myrelid and Øresland,^30^ and Berghog *et al*^6^ recommended routine use of mesalazine suppositories after colectomy, which may increase the number of patients eligible for IRA by reducing proctitis and increasing rectal compliance.

### Fecundity and sexual function

As the onset of UC is most commonly before the age of 30 years,^33^ fecundity and sexual function are important considerations for both male and female patients. Despite this, only two papers in this review commented on sexual function^7,23^ and none on fertility, showing that this is an area that requires further assessment. Tonelli *et al* reported 1% of patients with sexual restrictions after IRA compared with 2% after IPAA,^23^ and da Luz Moreira and Lavery reported 14% and 12% of patients with restrictions after IRA and IPAA respectively.^7^

In a non-systematic commentary on the literature, Myrelid and Øresland^30^ noted papers showing no change in fecundity after IRA but a significant decrease after IPAA, including a meta-analysis showing an increase in infertility rates after IPAA ranging from 20% to 63%.^34^ The mechanisms for this include damage to pelvic nerves as well as adhesions or chronic pelvic sepsis after a leak causing occlusion of fallopian tubes. The adhesion risk is felt to be reduced in the era of laparoscopic surgery,^35^ which supports the recommendation in the 2020 UK consensus standards for inflammatory bowel disease (IBD) that IBD surgery should be performed by specialist surgeons.^3^

### Postoperative complication rates

For this literature review, we considered papers only from 2000 onwards in order to represent current standards of surgical care most closely. Generally, IRA is considered to be a safer, less morbid and less complex operation than IPAA.^7^

Eight papers discussed postoperative complications after IRA.^6,7,14–17,20,23^ Five papers reported on anastomotic leak rate, which ranged from 1.6% to 5.4%.^7,15–17,23^ Four papers reported on mortality after IRA, with two noting comparable rates of 0%^7^ and 0.9%,^17^ and two giving much higher rates of 7%^6^ and 30%^20^ but both of these stating that the deaths were unrelated to the surgery. The mean overall morbidity rate for IRA was 17.2% (range: 4–28%).^7,14,16,17,23^ This compares with an overall morbidity rate for primary IPAA of 23% given in the 2017 *Ileoanal Pouch Report*.^36^

## Discussion

UC affects young people and can have a significant impact on quality of life.^33^ The decision about further surgery is different for each individual patient and can change at different stages in their lives. This systematic review of the literature seeks to provide a comprehensive discussion of one of the reconstructive operations, the IRA, with focus on those areas that matter most to patients and surgeons: cancer risk, failure rate, bowel function, fertility, sexual function and postoperative complications. It also highlights where decision making can be further individualised (e.g. for patients with dysplasia in the resected colon having higher cancer risks or patients with PSC having higher failure rates).

The cancer risk remains present until the rectum is fully removed and the risk is not fully eliminated even with pouch surgery although mucosectomy has been shown to reduce the risk even further.^29^ All the papers reviewed demonstrated a cancer risk of <5% at ten years after IRA and this risk can be partially mitigated with annual surveillance sigmoidoscopy.^23^ Likewise, the median failure rate of 21% at ten years has been highlighted previously as a reason to avoid IRA but this also represents nearly 80% of patients with a functioning IRA at ten years.

IRA appears to be a safe procedure^7^ with generally lower morbidity than pouch surgery. It is important to note that previous IRA does not preclude successful subsequent pouch surgery.^19^ IRA could therefore provide a period of life lived without the risks of pelvic dissection, sepsis, pouch or stoma, particularly relevant for this young cohort of patients who may be undergoing higher education, building a career or relationships, or starting a family. There may also be a role for patients with indeterminate colitis, to delay pouch surgery that could be severely compromised by a later diagnosis of Crohn's disease.

In terms of function, IRA scores slightly better than IPAA with regard to bowel frequency and continence, and less well on urgency and ongoing need for medication. The relative merits of either of these compared with life with a stoma is dependent on each patient's experience and priorities.

### Study limitations

One limitation of this review is that although the papers were all published after 2000, the data are often taken from patients treated before 2000 and are consequently still not fully representative of current practice. There is a publication bias with all the papers considered as results tend to be published by advocates of the procedure who have good outcomes.

There is also a paucity of data on bowel and sexual function, quality of life and the impact of the laparoscopic era on fertility. All of this is essential information for a quality-of-life operation such as an IRA or IPAA. The planned multicentre prospective comparison of these two procedures with the CRUISE (Colectomy Reconstruction for Ulcerative colitis In Sweden and England) study will add invaluable information to the discussion.^37^ Furthermore, the overall number of patients choosing to undergo reconstructive surgery is reducing and those eligible for IRA represent a small cohort of these patients.

## Conclusions

While the gold standard for restoration of gastrointestinal continuity remains IPAA in both UK and European guidelines, we need to ensure that patients have a comprehensive discussion about the risks and benefits of all their options, including IRA. This literature review summarised the evidence to aid this discussion for surgeons and their patients.

## Acknowledgements

The initial search results for this review were previously presented orally at the Association of Coloproctology of Great Britain and Ireland (ACPGBI) South West Chapter meeting in March 2022 and as a poster at the ACPGBI annual meeting in July 2022.
